# PGE2 upregulates the Na^+^/K^+^ ATPase in HepG2 cells via EP4 receptors and intracellular calcium

**DOI:** 10.1371/journal.pone.0245400

**Published:** 2021-01-14

**Authors:** Rawad Hodeify, Mohamed Chakkour, Reem Rida, Sawsan Kreydiyyeh

**Affiliations:** 1 Department of Biotechnology, School of Arts and Sciences, American University of Ras Al Khaimah, Ras Al Khaimah, United Arab Emirates; 2 Department of Biology, Faculty of Arts & Sciences, American University of Beirut, Beirut, Lebanon; Universidade Federal do Rio de Janeiro, BRAZIL

## Abstract

The Na^+^/K^+^ ATPase is a key regulator of the hepatocytes ionic homeostasis, which when altered may lead to many liver disorders. We demonstrated recently, a significant stimulation of the Na^+^/K^+^ ATPase in HepG2 cells treated with the S1P analogue FTY 720P, that was mediated through PGE2. The mechanism by which the prostaglandin exerts its effect was not investigated, and is the focus of this work. The type of receptors involved was determined using pharmacological inhibitors, while western blot analysis, fluorescence imaging of GFP-tagged Na^+^/K^+^ ATPase, and time-lapse imaging on live cells were used to detect changes in membrane abundance of the Na^+^/K^+^ ATPase. The activity of the ATPase was assayed by measuring the amount of inorganic phosphate liberated in the presence and absence of ouabain. The enhanced activity of the ATPase was not observed when EP4 receptors were blocked but still appeared in presence inhibitors of EP1, EP2 and EP3 receptors. The involvement of EP4 was confirmed by the stimulation observed with EP4 agonist. The stimulatory effect of PGE2 did not appear in presence of Rp-cAMP, an inhibitor of PKA, and was imitated by db-cAMP, a PKA activator. Chelating intracellular calcium with BAPTA-AM abrogated the effect of db-cAMP as well as that of PGE2, but PGE2 treatment in a calcium-free PBS medium did not, suggesting an involvement of intracellular calcium, that was confirmed by the results obtained with 2-APB treatment. Live cell imaging showed movement of GFP–Na+/K+ ATPase-positive vesicles to the membrane and increased abundance of the ATPase at the membrane after PGE2 treatment. It was concluded that PGE2 acts via EP4, PKA, and intracellular calcium.

## Introduction

The liver plays an important role in metabolic homeostasis that is highly dependent on cellular ionic balance. The latter is accomplished in hepatocytes, by the synchronized activity of various carriers and transporters [1]. The Na+/K+ ATPase or the Na+/K+ pump plays a key role in the control of the ionic intracellular milieu, a process that is needed for the regulation of metabolism, proliferation, differentiation and most importantly cell volume. It establishes the Na+ gradient needed for the transport of bile salts [2] and drives the activity of many other transporters like the Na+ /H+ exchanger and the Na+ -K + 2Cl cotransporter involved respectively in the regulation of intracellular pH and cell volume. Changes in hepatocyte’s cell volume are associated with changes in, bile flow and bile acid transport, cytoplasmic and endosomal pH, metabolism of carbohydrates, proteins and lipids, transcription and translation, and alteration of cytoskeleton components [1]. The activity of the pump is reduced in apoptosis [3] and increased in cellular proliferation [4]. An alteration in the Na+ -K + ATPase activity is thus expected to result in a derangement of many of the liver functions.

Emerging evidence provides a role for sphingolipids in liver diseases and hepatocellular death [5]. Sphingosine 1-phosphate (S1P) was reported to be involved in lipopolysaccharide induced liver injury and hydrophobic bile acid apoptosis [6, 7]. Recently, a novel sphingosine analog FTY720, was developed and approved by the US Food and Drug Administration as an oral treatment for relapsing forms of multiple sclerosis [8, 9]. FTY720 is rapidly phosphorylated in vivo by sphingosine kinase to FTY720-P which acts as an agonist at four S1P receptors namely S1P_1_, S1P_3_, S1P_4_, and S1P_5_. We have shown previously a significant increase in the activity of the Na^+^/K^+^ ATPase in HepG2 cells, treated with FTY720P for 2 hrs [10], that was mediated via S1PR3 and sequential activation of PKC, ERK, and NF-κB leading to a higher expression level of COX-2 enzyme and PGE2 release. However, the mechanism by which PGE2 modulates the ATPase activity remained unexplored and is the focus of this work. The aim of this study is to determine the process by which the prostaglandin alters the Na+/K+ ATPase activity.

## Materials and methods

### Materials

L-826266, SC-51089, BGC 20–1531 hydrochloride, and butaprost, were obtained from Cayman Chemical Company, Michigan, USA, while TSC 2510 was acquired from Tocris Bioscience, Bristol, United Kingdom.

Prostaglandin E2 (PGE2), Ouabain, Dulbecco’s Minimal Essential Medium (DMEM) with 4500mg/L Glucose and pyridoxine HCL, Trypsin-EDTA, Penicillin/Streptomycin, Fetal Bovine Serum (FBS), 10x Phosphate Buffered Saline (PBS) without magnesium and calcium, Adenosine 5’-triphosphate disodium salt (ATP), and anti-Na+/K+ ATPase α-1 Antibody, clone C464.6 were procured from Sigma, Chemical Co, St Louis Missouri, USA.

Protease inhibitor cocktail tablets were purchased from Boehringer Mannheim, Germany. Biorad protein assay reagent, nitrocellulose membranes and western blotting luminol and peroxidase (Clarity TM Western ECL Substrate) reagent were obtained from Biorad, California, USA. The Human hepatocellular carcinoma cell line, HepG2, was purchased from ATCC.

GFP-tagged Na+/K+ ATPase α-1 plasmid was a generous gift from Sznajder Laboratory, Northwestern University. The membrane marker, mCherry-Mem, was a gift from Catherine Berlot (Addgene plasmid # 55779 Live cell analysis of G protein beta5 complex formation, function, and targeting. Yost EA, Mervine SM, Sabo JL, Hynes TR, Berlot CH. Mol Pharmacol. 2007 Oct;72(4):812–25. Epub 2007 Jun 27. 10.1124/mol.107.038075). ViaFect™ Transfection Reagent was procured from Promega.

All other chemicals were purchased from Sigma, Chemical Co, St Louis Missouri, USA.

### Culture of HepG2 cells

HepG2 cells at passages 28–35 were grown in DMEM supplemented with 1% penicillin (100μg/ml) and streptomycin (100μg/ml) and 10% FBS at a density of 120,000 cells/ml on 100 mm culture plates. The cells were kept in humidified incubator (95% O2, 5% CO2) at 37°C and treated at 85–90% confluence after an overnight starvation.

### Treatment of HepG2 cells

Following starvation, HepG2 cells were treated for 2 hours with 100 nM PGE2. The cells were then washed with PBS buffer (pH = 7.4), scraped in lysis buffer in presence of protease inhibitors, homogenized for 30 seconds using PRO Homogenizer at maximum speed (around 30,000 rpm), and spun for 30 min at 20000g and 4°C. Proteins in the cell lysates were quantified colorimetrically at a wavelength of 595 nm using the Bradford method and then the homogenates were used to assay for the ATPase activity.

### Determination of the type of EP receptors involved

Receptor agonists and antagonists were used to identify the type of EP receptors mediating the effect of PGE2 on the pump. Cells were treated for 2 hrs with PGE2 (100nM) in the simultaneous presence of a selective blocker of each of the four types of EP receptors, namely PF-04418948 (1 μM, EP2 antagonist), L-798106 (10μM, EP3 antagonist), SC-19220 (100 μM, EP1 blocker) and BGC 20–1531 (10 μM, EP4 blocker). All antagonists were added 30 min before PGE2. The results were confirmed by studying the effect of specific agonists to the suspected receptors: TCS 2510 (1 μM, EP4 agonist).

### Involvement of calcium

To determine if calcium is a second messenger of PGE2, HepG2 cells were treated with the prostaglandin in the simultaneous presence of BAPTA-AM(20nM), a chelator of intracellular calcium, or in the simultaneous presence of 2-aminoethoxydiphenyl Borate (2-APB) (60 μM), a blocker of IP3 channels.

### Membrane fractionation

For membrane isolation, normal HepG2 cells not expressing the GFP-tagged Na^+^/K^+^ ATPase were used and treated with PGE2. At the end of the incubation period, the cells were washed with PBS and 500μl of HEPES buffer (20 mM HEPES (pH 7.4); KCl (10mM); MgCl2 (2 mM); EDTA (1 mM); EGTA (1 mM); DDT (1mM), protease inhibitors were added to each well on ice. The cells were then scraped, and passed through a 26-gauge needle 10 times, then through a 27-gauge needle for an additional ten times, and left on ice for 20min. The lysed cells were then spun at 720g for 5 min. The obtained supernatant containing cytoplasm, membranes and mitochondria was then subjected to a 5 min centrifugation at 10,000g. The new supernatant obtained was spun in an ultracentrifuge at 100,000g for one hour. The resultant pellet was washed with the HEPES buffer, re-suspended in the same buffer, passed through a 25-gauge needle and spun again at 100,000g for 45 min. The pellet containing the membranes was re-suspended in HEPES buffer and used for the assessment of the Na^+^/K^+^ ATPase activity and expression. All centrifugation steps were conducted at 4°C.

### Na^+^/K^+^ ATPase assay

Cell homogenates or crude membranes were diluted to a protein concentration 0.5 μg/μl with histidine buffer (pH 7.4, 150mM) and incubated for 15 min at room temperature with 1% saponin added at a ratio of 1:4, in presence of phosphatase inhibitors (2.7 mM pyrophosphate, 2.7 mM glycerophosphate). Aliquots were then taken from each sample and incubated at 37°C and for 30 min in histidine buffer containing NaCl (121.5mM), KCl (19.6 mM), MgCl2 (3.92 mM), adenosine tri-phosphate (2.94 mM), in presence or absence of ouabain (1.47 mM), a specific inhibitor of the ATPase. When ouabain was absent, it was replaced with water. The reaction was stopped by addition of 50% trichloroacetic acid at a ratio of 1:10 (v/v) and the samples were spun at 3000g for 5 min. The amount of inorganic phosphate liberated in the supernatant (μg/mg protein /min) was measured colorimetrically at 750 nM according to the method of Taussky H, Shorr [11], and reported as a percentage of the control value. The measured activity ranged between 13 and 57 nanomoles Pi/mg protein /min.

### Trafficking assay of the Na+/K+ ATPase

To determine if changes in the activity of the Na^+^/K^+^ ATPase are due to changes in its cellular trafficking, the treatment of HepG2 cells with PGE2 was conducted on ice, and the activity of the ATPase was assayed in the membrane fraction and cell homogenate.

### Involvement of PKA in the effect of PGE2 on the Na^+^/K^+^ ATPase

Since EP4 receptors are coupled to Gs proteins which activate adenylate cyclase and lead to cAMP production and PKA activation, and since PKA has been reported previously to modulate the ATPase activity, the involvement and the positioning of PKA in the signaling pathway were investigated. HepG2 cells were treated with PGE2 (100nM, 2hours) in presence of an inhibitor of PKA namely RpcAMP (30 μM) added 20 min prior to the prostaglandin, or with dbcAMP (10 μM, 2hrs), a cell permeable cAMP analogue. To position calcium with respect of PKA, the cells were treated with db-cAMP in presence of BAPTA-AM (20nM), which was added 20 min before.

### Western blot analysis

Ten micrograms of membrane proteins or 40μg of homogenate proteins, obtained from normal cells not expressing GFP-tagged Na^+^/K^+^ ATPase, were resolved on 10% SDS polyacrylamide gels and transferred to a nitrocellulose membrane. The membranes were then blocked, and subsequently incubated overnight at 4°C with a Na^+^/K^+^ ATPase antibody, followed by an incubation with goat anti-rabbit HRP conjugated IgG secondary antibody for 1 hour at room temperature. The signal was detected by chemiluminescence using the Clarity ECL Substrate. The intensity of the signal was detected using a ChemiDoc^TM^MP imaging system.

### Cell transfection and imaging

To measure surface Na+/K pump, a GFP-tagged Na^+^/K^+^ ATPase α-1 plasmid was used (a generous gift from Sznajder Laboratory, Northwestern University) and the membrane marker, mCherry-Mem, a gift from Catherine Berlot (Addgene plasmid # 55779). HepG2 cells were seeded on Poly-D-Lysine coated glass-bottomed dishes (MatTek) one day before co-transfection with 1.5 μg GFP-tagged Na,K-ATPase α1 plasmid DNA and 1.5 μg mCherry-Mem plasmid DNA using ViaFect™ Transfection Reagent. After 48 hours of transfection, cells were treated for 2 hours with 100 nM PGE2 before imaging with confocal microscope Zeiss LSM710. Images were processed with ImageJ (National Institutes of Health) and Adobe Photoshop CS3. To quantify the relative membrane expression of GFP-Na,K-ATPase in control and after PGE2 treatment, single cells positive for GFP and mCherry-Mem were selected and fluorescence ratio of green/red was determined in individual cells.

### Statistical analysis

Results are reported as mean ± SEM, and tested for statistical significance using a one-way analysis of variance followed by a Tukey-Kramer multiple comparison test using GraphPad InStat 3.

## Results

### PGE2 acts via EP4 receptors

**Fig 1:**
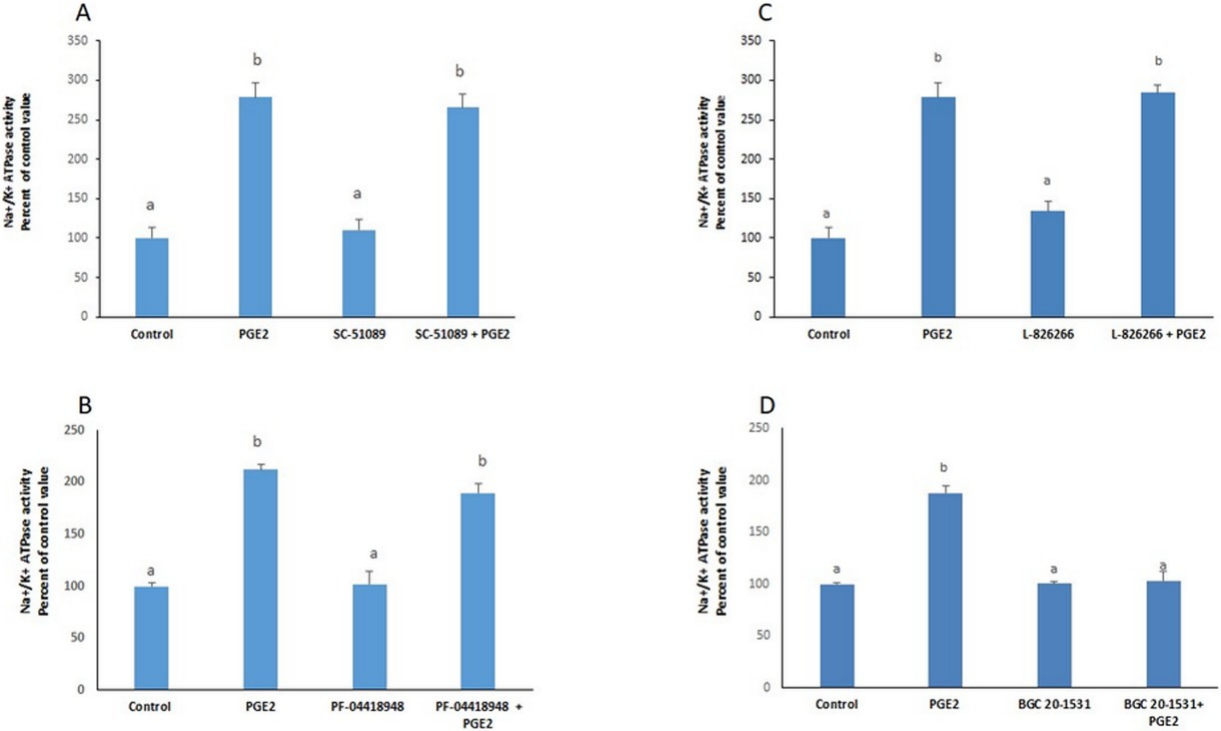
PGE2 acts via EP4 receptors. Effect of PGE2 (100nM, 2hours) on the activity of the Na^+^/K^+^ ATPase in presence of (**A**) SC-19220 (EP1 antagonist); (**B**) PF-04418948 (EP2 antagonist); (**C**) L-798106 (EP3 antagonist); (**D**) BGC 20–1531 (EP4 antagonist), added 30min before PGE2. Values are means ± SEM of 3 observations. Bars not sharing a common letter are significantly different from each other at p< 0.01.

**Fig 2:**
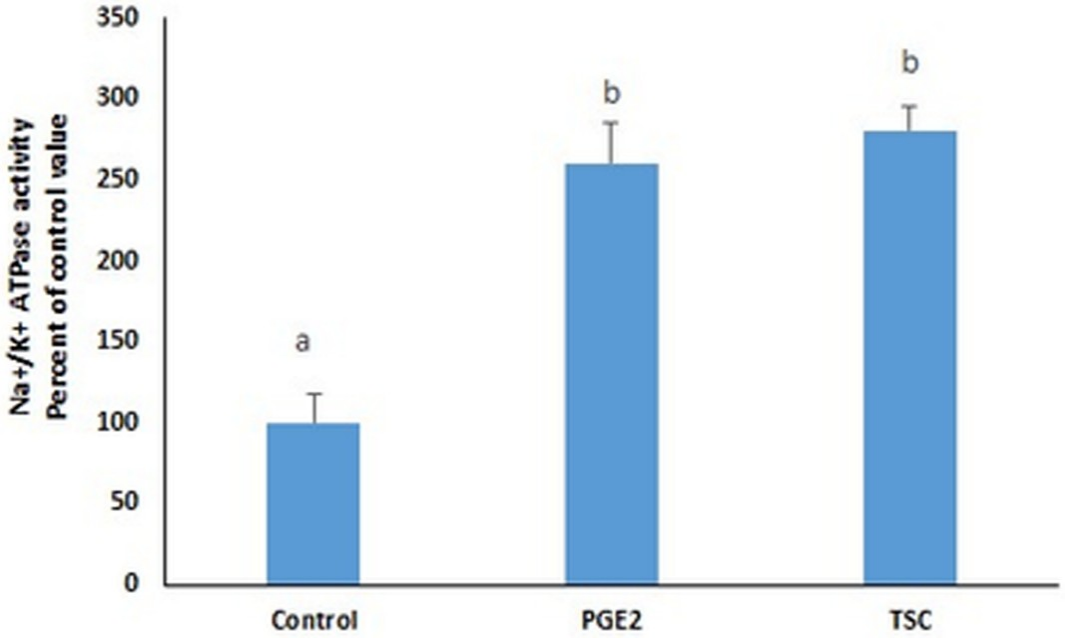
TCS-2510, an EP4 agonist, increases the activity of the Na^+^/K^+^ ATPase. Values are means ± SEM of 3 observations. Bars not sharing a common letter are significantly different from each other at p< 0.01.

PGE2 exerted a significant increase in the activity of the Na^+^/K^+^ ATPase that was still manifested in presence of blockers of EP1(SC-19220), EP2 (PF-04418948) and EP3 (L-798106), but disappeared completely in presence of BGC 20–1531, an antagonist of EP4 receptors (Fig 1), suggesting that PGE2 acts via EP4 receptors. The results were confirmed by the significant stimulation observed with TC 2510, a specific agonist of EP4 receptors (Fig 2).

### PGE2 increases the abundance of the Na^+^/K^+^ ATPase in the membrane

**Fig 3:**
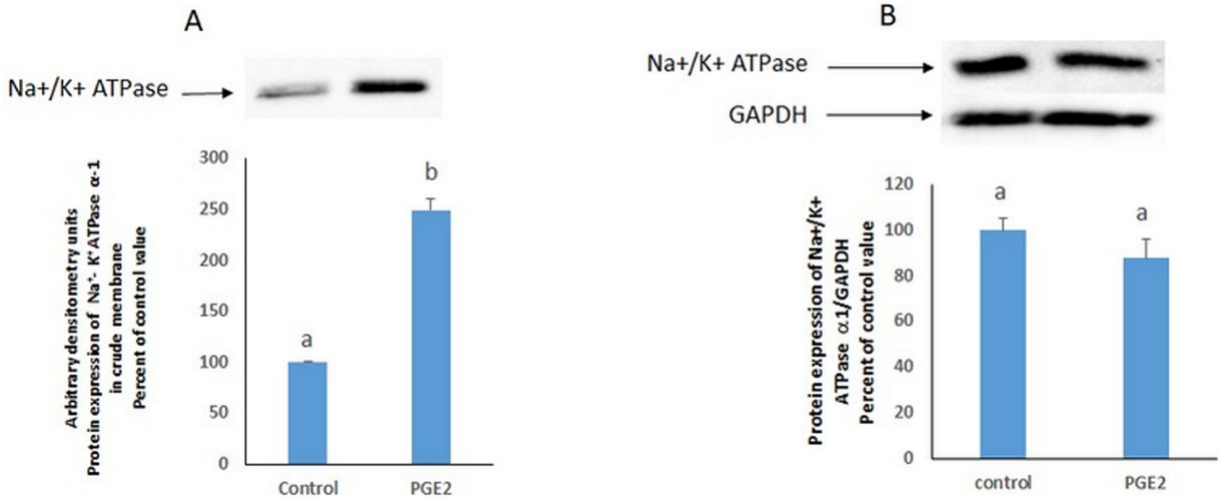
(A) PGE2 increases the protein expression of the Na^+^/K^+^ ATPase in a crude membrane preparation but (B) not in the whole cell homogenate. Values in (B) are normalized to GAPDH using Image lab software. The blots are representative of an experiment repeated 3 times. Values are means ± SEM of at least 3 observations. Bars not sharing a common letter are considered significantly different from each other p< 0.01.

Western blot analysis revealed no change in the protein expression of the ubiquitously expressed α1 subunit of the endogenous Na^+^/K^+^ ATPase in cell homogenates. However, in the crude cell membrane preparation obtained by cell fractionation, a significant increase in the ATPase expression was induced by PGE2 that was commensurate with the observed increase in its activity (Fig 3).

### Distribution of GFP- Na ^+^/K^+^ ATPase within the cells

**Fig 4:**
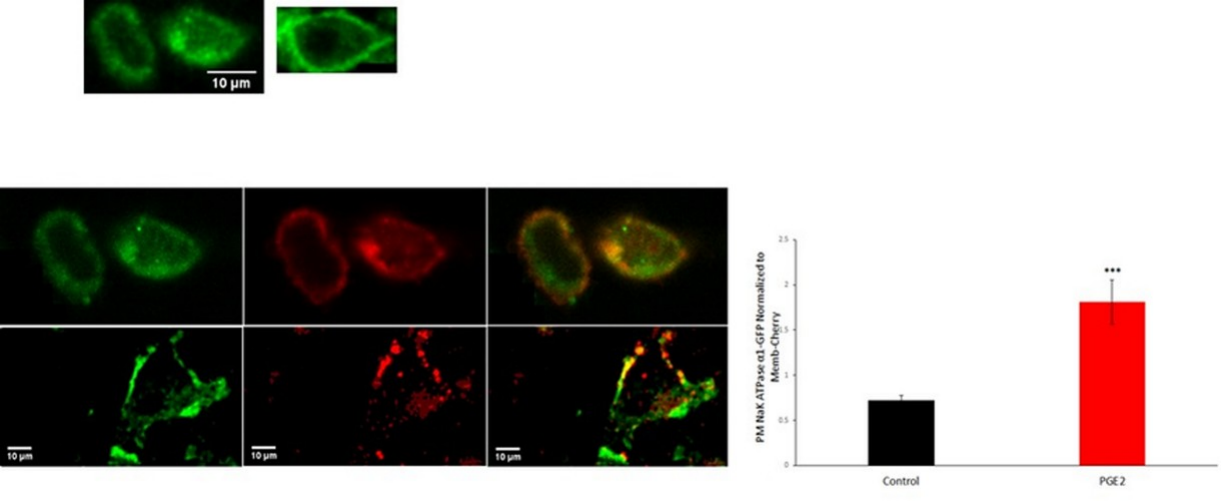
PGE2 increases the enrichment of Na^+^/K^+^ ATPase at plasma membrane. (A) Representative fluorescent images from HepG2 cells expressing GFP-tagged Na^+^/K^+^ ATPase α1 with or without PGE2. Arrows indicate plasma membrane enrichment of the pump. Scale bar: 10 μM. (B) Representative images of cells co-expressing GFP- Na^+^/K^+^ ATPase α1 and mCherry-Membrane marker under control or PGE2-treated conditions. Scale bar: 10 μM. (C) Quantification of plasma membrane associated ATPase normalized to mCherry-membrane marker. Values are means ± SEM (***P<0.001).

To further confirm the increase in Na^+^/K^+^ ATPase abundance at plasma membrane, enrichment of the Na^+^/K^+^ ATPase at the plasma membrane in HepG2 treated with PGE2 was tested by fluorescence imaging of GFP-tagged Na^+^/K^+^ ATPase α1 in the presence of a mCherry-membrane marker. Relative quantification of GFP/mCherry signal showed ~2.5-fold increase in PGE2-treated cells as compared to control cells (Fig 4). Time-lapse imaging on live cells illustrated the dynamic nature of the pump with GFP–Na^+^/K^+^ ATPase α1-positive vesicles budding off and fusing with the plasma membrane (S1A and S1B Movies).

### PGE2 induces trafficking of the Na^+^/K^+^ ATPase from intracellular stores to the cell membrane

**Fig 5:**
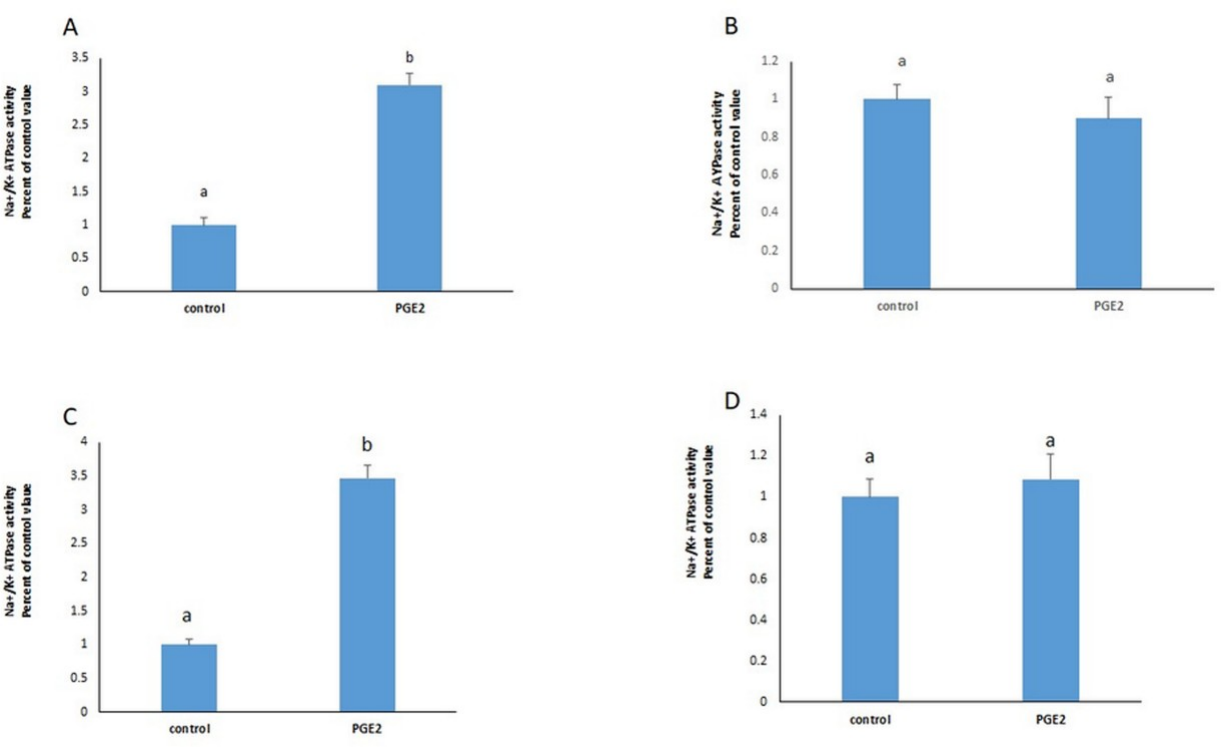
The stimulatory effect of PGE2 on the Na^+^/K^+^ ATPase in cell homogenates (A) and membrane fractions (C) did not appear when the cells were treated on ice (B, homogenate & D, membrane fractions). Values are means ± SEM of 3 observations. Bars not sharing a common letter are significantly different from each other at p< 0.01.

PGE2 induced in the membrane fraction, an increase in the activity of the Na+/K+ ATPase (Fig 5C) similar to the one observed in cell homogenates (Fig 5A). The effect of the prostaglandin disappeared completely however, when the cells were treated on ice, in the absence of the energy needed for the trafficking of the ATPase (Fig 5C, homogenate & 5D, membrane fraction).

### Involvement of calcium in the response to PGE2

**Fig 6:**
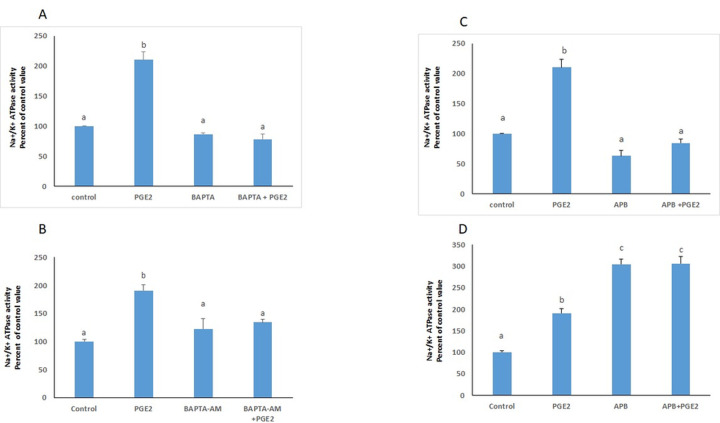
Calcium mediated the effect of PGE2. Effect of BAPTA-AM on the Na^+^/K^+^ ATPase activity in cells treated with PGE2 in (**A**) a calcium free PBS medium or (**B**) a calcium containing DMEM medium. Effect of 2-APB on the Na+/K+ ATPase activity in cells treated with PGE2 in (**C**) a calcium free PBS medium or (**D**) a calcium containing DMEM medium. Values are means ± SEM of 3 observations. Bars not sharing a common letter are significantly different from each other at p< 0.01.

The involvement of calcium in the response to PGE2 was investigated by studying the effect of the prostaglandin on the Na^+^/K^+^ ATPase in cells treated in a calcium-free phosphate buffered saline (PBS) or in a normal medium, in the simultaneous presence of BAPTA-AM, a calcium chelator. In both cases, the absence of free intracellular calcium abrogated the effect of PGE2. In presence of 2-APB, a blocker of IP3 channels, the PGE2 induced increase in the ATPase activity did not appear when cells were treated in calcium-free PBS, but was significantly enhanced when the cells were treated in normal medium (Fig 6).

### PKA is involved in the response to PGE2

**Fig 7:**
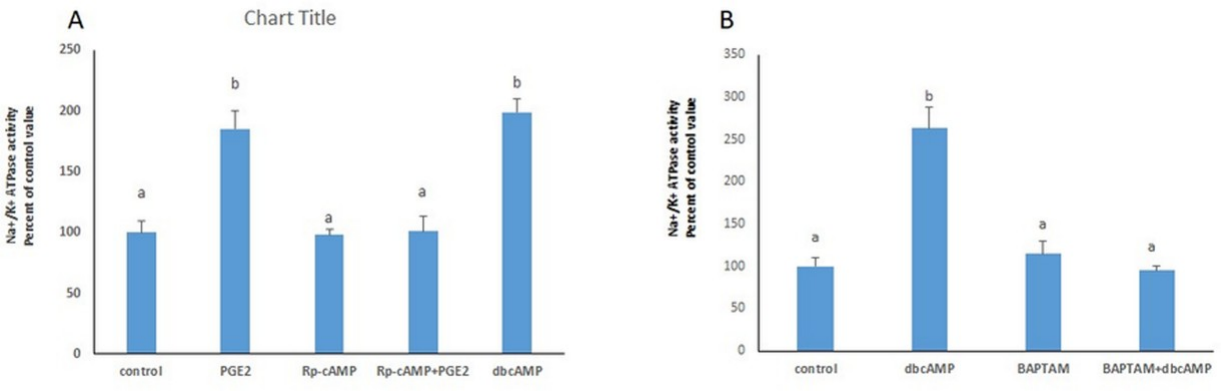
PGE2 acts via PKA and calcium. (A) The stimulatory effect of PGE2 (100 nM) on Na^+^/K^+^ ATPase disappeared in the presence of the PKA inhibitor (Rp-cAMP: 30 μM), added 20 min before PGE2, and was imitated by dbcAMP (10 μM). (B) The effect of PGE2was not manifested in presence of BAPTA-AM (20nM). Values are means ± SEM. N = 3. Bars not sharing a common letter are statistically significant from each other at p<0.001.

The stimulatory effect of PGE2 on the Na^+^/K^+^ ATPase disappeared in the presence of the PKA inhibitor Rp-cAMP (Fig 7A), while treating the cells with dbcAMP (Fig 7A), a PKA activator, resulted in a similar significance increase in the ATPase activity.

### Calcium acts downstream of PKA

The enhanced Na+/K+ ATPase activity induced by dbcAMP was abrogated in presence of the calcium chelator BAPTA-AM, inferring that the stimulatory effect is mediated through intracellular calcium (Fig 7B).

## Discussion

Prostaglandin E2 is a primary bioactive prostanoid derived from cyclo-oxygenase mediated metabolism of arachidonic acid. PGE_2_ exerts a broad range of physiological effects by acting through specific receptors to maintain the local homeostasis in the body [12]. The effect of PGE2 at two hours, on the Na^+^/K^+^ ATPase in HepG2 cells was previously studied and found to be dose dependent, with an inhibition appearing at low concentrations, and a stimulation induced at a 10nM concentration and above [10]. Four different types of PGE2 receptors have been identified and are known as EP1, EP2, EP3, and EP4 [13]. All of them are coupled to G-proteins. EP2 and EP4 are coupled to Gs, EP1 is linked to Gq, and EP3 to Gi. Activation of each of these G proteins activates a specific signaling pathway [13]. In this work, blocking EP1, EP2 and EP3 receptors with respectively SC-19220, PF-04418948 and L-798106 did not abolish the effect of PGE2 on the ATPase. However, in presence of BGC 20–1531, a blocker of EP4, the stimulatory effect of PGE2 disappeared completely. The results suggest that PGE2 enhances the ATPase activity by binding and activating EP4 receptors which are coupled to Gs and thus activate adenylate cyclase generating cAMP and activating PKA.

The PGE2-induced increase in the activity of the Na^+^/K^+^ ATPase may be due to an increase in the specific activity of the enzyme or an increase in its abundance in the membrane. To clarify this issue we resorted to fluorescence imaging to determine changes in the abundance of GFP-tagged Na^+^/K^+^ ATPase α1 subunits in the membrane. Time-lapse imaging illustrated the dynamic recycling and significant enrichment of the pump upon PGE2 treatment, at the cell membrane. Similarly, endogenous Na^+^/K^+^ ATPase expression at plasma membrane as well as activity were enhanced by PGE2 in the membrane fraction (Figs 3 and 6). The results revealed an enhanced movement of the Na^+^/K^+^ ATPase to the cell membrane and an increase in its membrane abundance that was supported by the disappearance of the PGE2 effect under low temperature. Western blot analysis showed that PGE2 did not alter the protein expression of the ATPase in the whole cell but induced a significant increase in its expression only in the membrane, an increase that was commensurate with the observed increase in its activity. These findings suggest that the enhanced expression of the α1 subunit in the membrane is not the result of an increase in its synthesis but rather a result of a modulation of its trafficking. It remains possible that the enrichment at membrane by PGE2 is due to enhanced exocytosis and/or fusion of Na^+^/K^+^ ATPase positive vesicles or decreased endocytosis. Deciphering the exact mechanism requires kinetic studies of exocytosis and endocytosis of Na^+^/K^+^ ATPase in the presence of PGE2. However, the data reported here demonstrating the association of enrichment at membrane with intracellular calcium and its abrogation in cells treated on ice, may suggest that increased abundance of the pump at membrane is due to enhanced exocytosis.

Because calcium is known to play a role in cellular trafficking [14] its involvement in the effect of PGE2 was investigated. In presence of BAPTA-AM, a permeant calcium chelator, the PGE2 induced increase in the ATPase activity disappeared completely, inferring that PGE2 acts via an increase in intracellular calcium which can come from intracellular stores or from outside. To investigate the involvement of extracellular calcium, the effect of PGE2 was studied in cells treated in a calcium-free PBS buffer. The data revealed a similar stimulation to the one observed in a normal incubation medium, indicating that the calcium source is intracellular. To confirm this hypothesis, the effect of 2-APB, an inhibitor of IP3 receptors, was investigated. Cells treated in PBS with PGE2 and 2-APB simultaneously did not show any change in their Na^+^/K^+^ ATPase activity, supporting thus the hypothesis. However, when the cells were treated in a calcium-containing normal medium, PGE2, in presence of 2-APB enhanced further the ATPase activity, probably by activating calcium influx, as reported in other works demonstrating activation of calcium entry by 2-APB, when used at similar concentrations to the one used in our study, through stimulation of TRPV and Orai3 channels [15–17]. Notably, 2-APB alone enhanced the activity of the ATPase to a higher extent than PGE2, a process that could be ascribed to a higher increase in calcium influx.

PGE2 was found to modulate the ATPase activity via EP4 receptors. These receptors are coupled to Gs proteins that activate adenylate cyclase, increase cAMP levels leading to activation of cAMP-dependent protein kinase (PKA) [18, 19]. Kinases are known modulators of the activity of the Na+/K+ ATPase. Their effect however is cell type dependent and varies from activation to inhibition. PKA was reported to reduce the activity of the Na^+^/K^+^ ATPase in the loop of Henle and collecting duct (Kiroytcheva 1999 Effect of cAMP on the activity and the phosphorylation of Na^+^,K^+^-ATPase in rat thick ascending limb of Henle [20] and enhance it in the proximal convoluted tubules [21]. This response seems to be mediated, at least in part, by phosphorylation of Ser 943 of the α-subunit suggesting that a phosphorylation/dephosphorylation event may dynamically regulate the activity of the pump. PKA may however stimulate the ATPase via pathways that do not entail its phosphorylation but rather its trafficking between an intracellular pool and the plasma membrane. Such a process has been reported in the proximal convoluted tubule whereby PKA increased the number of Na+-K+ ATPase units in the plasma membrane [22] and has been observed also in this work.

Activating PKA with db-cAMP resulted in a significant increase in the activity of the Na+/K+ ATPase that was dependent however on the presence calcium released from intracellular stores (Fig 7). A role of PKA as a regulator of calcium release has been already reported in the literature. Several studies demonstrated in hepatocytes, potentiation of IP3-induced calcium release by PKA [23, 24], and the specific phosphorylation of the major calcium channel isoform, IP3R2, at Ser937, promoting IP3R gating [25]. In addition, PKA phosphorylation of IP3 receptors has been associated with an increase in IP3 binding affinity [26] and recruitment of IP3Rs into functional Ca2+ stores [24].

## Conclusions

It can be concluded that in HepG2 cells, the PGE2- induced stimulation of the Na^+^/K^+^ ATPase is mediated through EP4 receptors and is dependent on PKA activation and calcium release from intracellular stores. The observed enrichment of the plasma membrane in the ATPase, is probably the main factor behind the enhanced activity.

## Supporting information

S1 Raw image (TIF)

S2 Raw image (TIF)

S1 Movie Time-lapse imaging of GFP–Na^+^/K^+^ ATPase α1. HepG2 cells transiently expressing GFP–Na+/K+ ATPase α1 untreated (A) or treated with PGE2 (B) were imaged by confocal microscopy with frames collected every 1 sec. Movie display rate is 5 frames per second.
(ZIP)
