# Supplementary figures and images for: PGE2 upregulates the Na+/K+ ATPase in HepG2 cells via EP4 receptors and intracellular calcium
Source: PLoS One. 2021 Jan 14;16(1):e0245400. doi: 10.1371/journal.pone.0245400 (PMC7808645; doi:10.1371/journal.pone.0245400)

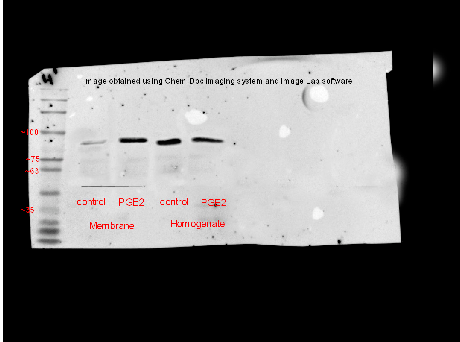

Supplement: S1 Raw image — (TIF) [file pone.0245400.s001.tif]

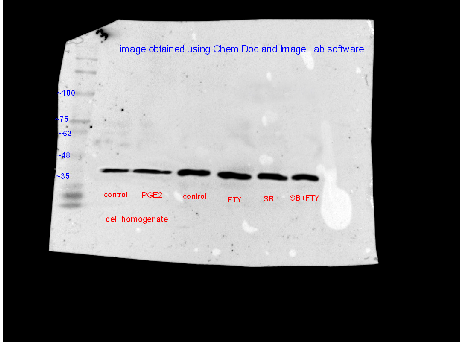

Supplement: S2 Raw image — (TIF) [file pone.0245400.s002.tif]
